# Proceedings of the Quarantine Convention

**Published:** 1857-07

**Authors:** 


### PROCEEDINGS OF THE QUARANTINE CONVENTION.

(Condensed from the published Report.)

The Quarantine Convention met in the Supreme Court Room, Philadelphia, May 3, 1857, and after a temporary organization, was addressed by Dr. Wilson Jewell, who stated the object of the meeting, read an invitation from the Philadelphia Board of Health, and on behalf of the Board, welcomed the delegates to Philadelphia.

Dr. Jewell then concluded his opening address with a brief sketch of the quarantine regulations in force at the different sea-ports of the United States.

The names of seventy-three delegates, from nine different States, were then read.

A nominating committee of one from each State having been appointed, and rules of order adopted, on motion of Mr. Haswell, of New York, a committee of five was appointed by the Chairman, to report the rule of representation by which the Convention was to be governed.

The chair appointed Mr. Haswell, of New York; Dr. Condie, of Philadelphia; Dr. Jones, of New Orleans; Mr. Quincey, of Baltimore; and Alderman James, of Boston.

The Committee on Nominations reported the following delegates for election, as permanent officers of the Convention:

*President.*—Dr. Wilson Jewell, of Philadelphia.

*Vice-Presidents.*—Dr. E. H. Barton, of New Orleans, and the Hon. Alex. H. Rice, of Boston.

*Secretaries.*—Dr. Edward Hartshorne, of Philadelphia, and Homer Franklin, Esq., of New York.

The nominations having been unanimously confirmed, the Convention was permanently organized, with the President, Dr. Jewell, in the chair.

After the reading of various invitations, on motion of Dr. Askew, of Delaware, it was

*Resolved,* That a Committee of seven be appointed to prepare and arrange business for the Convention.

Whereupon the Vice-President appointed Dr. Askew, of Delaware; Dr. Kemp, of Baltimore; Dr. Barton, of Louisiana; Dr. Condie, of Philadelphia; Dr. Hayward, of Boston; Dr. Parsons, of Rhode Island; and Dr. Selden, of Virginia.

The Committee to determine the rule of voting, then reported the following resolution:

Upon all questions involving the final decision or recommendation of this Convention, each body represented shall have one vote; but on all matters occurring in the current business of the Convention, each delegate shall have a voice.

A communication from Dr. Sterling, of New York, was referred to the Committee on Business.

On motion of Dr. Askew, Messrs. Homer Franklin, of New York, and J. H. Diehl, of Philadelphia, were added to the Committee on Business.

The Committee then offered a partial report and asked leave to sit again.

SECOND DAY.

The Convention having been called to order by the President, the roll was called, and fifty-four delegates answered to their names.

A letter from Dr. Thompson, of New York, was read.

The Committee on Business then presented a partial report printed, the resolutions contained in which formed the subjects of discussion throughout the day.

[We deem it unnecessary to insert the numerous suggestions, amendments, &c., offered by the various delegates during this and the succeeding day, but will content ourselves with the Report as finally adopted, premising that the Convention resolved to meet again next year at Baltimore, and that its name be changed to THE QUARANTINE AND SANITARY CONVENTION.]

The Report from the Committee on Business, as amended, reads as follows:

Whereas great interest has been awakened in the questions pertaining to commercial intercourse among the nations of the earth, and to the close relations, under various circumstances, of the health of communities to the regulations which affect this intercommunication; and inasmuch as there is great diversity and irregularity in the rigor of enactment which characterizes the legislation of different bodies upon this subject; therefore be it

*Resolved*, That it is expedient that the system of Quarantine Regulations be revised, and that correct principles, as far as scientific research and observation have developed them, should be the basis of future enactments, to the end that a uniform code, as far as practicable, may be secured in all our ports.

*Resolved*, That the following propositions be regarded as the sentiment of this Convention.

1. There are certain diseases which may be introduced into a community by foul vessels and cargoes, and diseased crews and passengers.

2. These diseases are small-pox, and under certain circumstances, typhus fever, cholera, and yellow fever.

3. When the latter diseases are introduced in this manner, their action is limited to individuals coming within their immediate influence, and cannot become epidemic or endemic, unless there exist in the community the circumstances which are calculated to produce such disease independent of the importation.

4. That the circumstances alluded to, consist in vitiated states of the atmosphere, from local causes, in connection with peculiar meteorological conditions.

5. Efficient sanitary measures, including quarantine, will in most cases prevent the introduction of these diseases, and may, at any rate, disarm them of their virulence, and prevent their extension when introduced.

6. The present quarantine regulations, in operation in most of our States, are inefficient, and often prejudicial to the interests of the community.

7. Disease may be introduced: 1st, by a foul vessel, especially when proper measures are not taken to keep the hold free from stagnant and putrid bilge-water; and more particularly when there exist in the hold droppings or drainage from putrescible matters which are allowed to penetrate and remain between the timbers of the ship. 2d. By cargoes consisting, in whole or in part, of rags, cotton, or like porous substances, shipped from ports at which any malignant epidemic or endemic disease of a contagious or infectious character prevailed at the time when the vessel was loaded. 3d. By the filthy bedding, baggage, and clothing of immigrant passengers, particularly when these are crowded together in insufficient quarters, although the passengers themselves may be free from any actual disease. 4th. By the air that has been confined during the voyage in closely sealed and ill-ventilated holds. 5th. By squalid and diseased passengers landed and crowded together in unhealthy neighborhoods, or in small and ill-ventilated dwellings. 6th. By passengers and crews, who are actually laboring under, or infected with any positively contagious disease; their bedding, clothing, and baggage.

8. To prevent, therefore, the introduction of disease from the several causes enumerated, the necessity is apparent of providing a system by which all parts of a vessel may be ventilated during a voyage; and for the careful inspection of all vessels immediately upon their arrival, and before they are allowed to come up to the wharves of a city, for the landing of their passengers and discharge of their cargoes. No vessel, arriving between the 1st of May and the 1st of November, should, in fact, be admitted to a port, until her hold is freely and fully ventilated, nor until the bilge-water is entirely removed.

9. Provision should be made for the immediate landing of all those portions of the cargo of a vessel, and the baggage and clothing that may be judged capable of generating or communicating disease, and for their proper purification, at such places and under such regulations as shall preclude all danger of their exerting a morbific influence, either immediately, or upon their subsequent admission into the city.

10. Provision should be made also for the immediate landing of all such persons from on board of vessels as they arrive, and their due and comfortable accommodation and treatment, until such time as they can be taken charge of, and properly cared for by their friends.

11. In the case of a ship-load of squalid passengers, or those strongly predisposed to disease, their clothing, beds, and other effects, should be at once subjected to a thorough ventilation and purification; and, upon their landing, adequate measures should be adopted to prevent them from crowding together in confined, unhealthy, and ill-ventilated dwellings and localities.

12. When a vessel arrives in a foul condition, or on board of which disease has prevailed during the voyage, after her crew and passengers have been removed from her, she should be subjected to a thorough process of cleansing and purification; for which purpose it may be necessary to discharge her cargo at a safe distance from the city, and to allow only such portions of it to be conveyed there as are incapable of creating disease, the residue being subjected to ventilation in such manner as shall prevent it from suffering damage and all unavoidable deterioration.

13. The carrying out of these provisions should be intrusted to a single officer, with such assistants as may be required to aid him in the performance of his functions.

14. This officer should be a regular physician, of unquestionable talents and experience, and possessed of great decision and rectitude of character.

15. His compensation should be sufficiently ample to enable him to devote his entire attention and energies, throughout the year, to the duties of his office.

16. While the power of removing him for incompetency, neglect, or other adequate cause, should be vested in some competent tribunal, his appointment should be based solely upon his capacity to fulfil satisfactorily his incumbent duties, and his continuance in office made dependent upon his faithful and skilful discharge of those duties.

17. To this officer should be intrusted the sole and entire decision, under certain general provisions established by law, as to the treatment required in the case of each vessel that shall arrive, and of its cargo, crew, and passengers, and to place it and these in a condition to prevent any danger of the introduction by them of disease; he, at the same time, being held to a strict accountability for the manner in which the discretionary power thus confided to him is executed.

18. As in every community a Board of Health is necessary to watch over its sanitary condition, and to prevent or remove all domestic sources of disease, this body would appear to be the one in which the power of appointing, and the general supervision of the official conduct of the Quarantine Physician may, with the greatest propriety, be invested.

19. In order to procure a uniformity in quarantine regulations throughout the several ports of the United States, the assembling of another, and probably several, conventions similar to the present one, will be required.

20. To provide for the assembling of such a convention in 1858, it is suggested that the President, Vice-Presidents, and Secretaries of this Convention, with a committee of one member from each State represented, be continued after our adjournment, as commissioners for the purpose of taking the necessary steps for the call of a Convention next year; provided, however, that their powers shall cease immediately upon the assembling and organization of the Convention of 1858.

21. A thorough examination should be made of all immigrants on their arrival; and if they are not protected against small-pox, they should be vaccinated.

22. We recommend that there should be attached to our Board of Health and Quarantine establishments stations for minute meteorological observations and vaccine establishments; and that records of these be published at stated periods, for the public benefit.

23. We advise the introduction of increased comforts for crews and passengers, and the ventilation and purification of vessels by a more effectual method.

On motion of Dr. Condie, it was

*Resolved*, That the vote of each delegation on the adoption of the Report of the Committee on Business be entered on the minutes.

The delegations voting in the affirmative were: Massachusetts—Boston Board of Health, Boston Port Physician (External Health), Boston Marine Hospital (Internal Health); Rhode Island—Providence Board of Health, Providence Medical Association; New York—Board of Health; New Jersey—Newark Board of Health, Camden Board of Health; Pennsylvania—Philadelphia Board of Health, Philadelphia Board of Trade, Philadelphia College of Physicians, Philadelphia County Medical Society; Delaware—Wilmington Board of Health, Medical Association of Wilmington; Maryland—Baltimore Board of Health, Baltimore Board of Trade, Baltimore Medical and Surgical Society, Baltimore Pathological Society. Those who voted in the negative were: Virginia—Norfolk Board of Health and Norfolk City Council. The delegations from the Common Council of New Orleans and the New Orleans Board of Health, reported each a tie vote.

On motion of Dr. Jones, of New Orleans, it was resolved—

1. That, in addition to the usual quarantine establishments, this Convention recommends the introduction of efficient means for removing all persons of limited means from infection, and for preventing the ingress of immigrants and other unseasoned people into ports and cities laboring at the time under pestilential diseases.

2. That in all cases where rumors and unauthorized reports indicate certain ports or cities as the seats of epidemic and pestilential diseases of the nature provided against, no such reports shall be the basis for action elsewhere, unless sustained by an official declaration of the Boards of Health, or other properly constituted authorities.

3. That all such Boards of Health and other public authorities shall be obligated to declare the existence of invasions of yellow fever and cholera, in their epidemic forms, as they may from time to time make their appearance in the localities under their control.

On motion of Dr. Snow, of Providence, it was then

*Resolved*, That we recommend the adoption of a complete, accurate, and uniform system of registration of births and deaths in all our cities, as a necessary accompaniment of efficient sanitary measures.

